# Targeting the CXCL12/CXCR4 pathway by an optimized derivative or EPI-X4 preserves chondrocyte function and offers a novel therapeutic approach in rheumatic diseases

**DOI:** 10.1186/s13075-025-03691-9

**Published:** 2025-12-02

**Authors:** Leonie Ruths, Hannah de Hesselle, Felix Haußner, Sofya Albers, Mirja Harms, Tobias Freitag, Jan Münch, Jana Riegger

**Affiliations:** 1 Department of Orthopaedics, Division for Biochemistry of Joint and Connective Tissue Diseases, University of Ulm, Ulm, 89081 Germany; 2 Department of Orthopaedic Surgery, University of Ulm, Ulm, 89081 Germany; 3 Department of Molecular Virology, Ulm University, Ulm, 89081 Germany

**Keywords:** Osteoarthritis, Therapy, CXCL12, CXCR4

## Abstract

**Background:**

The C-X-C motif chemokine 12 (CXCL12) and its receptor CXCR4 are pivotal in tissue regeneration and inflammation, yet their role in osteoarthritis (OA) remains ambiguous. However, it is assumed that the CXCL12/CXCR4 axis likely contributes to OA progression through subchondral bone-cartilage crosstalk. This study compares the efficacy of the CXCR4 inhibitors AMD31000 and novel endogenous peptide inhibitors in human cartilage and isolated chondrocytes (hAC).

**Methods:**

Human cartilage and hAC were obtained from OA patients undergoing arthroplasty. Expression of both CXCL12 receptors CXCR4 and ACKR3, were assessed by immunohistology and qRT-PRC. The effects of CXCR4 inhibitors, including AMD3100, EPI-X4, and its derivative EPI-X4 JM#21, were evaluated regarding cell viability, migration, chondrogenic and osteogenic differentiation, and proliferation of chondrocytes in presence of 200 ng/mL CXCL12.

**Results:**

The current data demonstrate that CXCR4 is significantly upregulated in OA cartilage and senescent chondrocytes, while ACKR3 expression remains largely unchanged. CXCR4 inhibition had no detrimental effects on chondrocyte viability, proliferation, or chondrogenic differentiation potential but effectively reduced CXCL12-induced cell migration. EPI-X4 JM#21 emerged as a potent CXCR4 antagonist and ACKR3 agonist, outperforming AMD3100 in suppressing chondrocyte migration. Although CXCR4 was significantly upregulated during osteogenic differentiation of hAC, the inhibition of the receptor had no effect on calcium deposition.

**Conclusions:**

These findings suggest that EPI-X4 JM#21 may represent potential alternative to AMD3100 for targeting the CXCL12/CXCR4 pathway in OA, warranting further in vivo validation.

**Supplementary Information:**

The online version contains supplementary material available at 10.1186/s13075-025-03691-9.

## Background

The C-X-C motif chemokine 12 (CXCL12), also termed as stromal cell-derived factor 1 (SDF-1), plays a regulatory role in various physiological processes, such as recruitment and differentiation of mesenchymal stem (MSCs) and progenitor cells. Accordingly, CXCL12 is considered as one of the most intensively investigated chemokines in terms of tissue regeneration, including fracture healing [1], meniscus [2] and hyaline cartilage repair [3]. Generally, CXCL12 can interact with two receptors: CXC chemokine receptor type 4 (CXCR4) and the atypical chemokine receptor 3 (ACKR3), formerly known as CXCR7. Although, CXCL12 signals through the G protein coupled receptor CXCR4, it exhibits a tenfold higher affinity for ACKR3. CXCL12/ACKR3 interaction does not activate G protein-dependent pathways, but mediates β-arrestin-2 recruitment and subsequent internalization of the chemokine [4]. Thus, ACKR3 plays a crucial role in modulating chemokine gradients and acts as a scavenger for CXCL12 and other chemokines. ACKR3 is therefore an important regulator of cell migration, angiogenesis, and immune responses [5]. Dysregulation has been implicated in various diseases, including cancer, cardiovascular disorders, and inflammatory conditions, where it contributes to tumor progression, vascular remodeling, and chronic inflammation [6–8].

The pro-regenerative properties of the CXCL12/CXCR4 axis in musculoskeletal tissues may be attributed to its chemotactic characteristics and the promotion of skeletal repair processes, e.g., by synergistic interaction with CC-chemokine ligand 5, transforming growth factor β1 (TGF-ß), and bone morphogenetic factor 2 (BMP-2) [9–12]. In accordance with this, it has been demonstrated that the chemotactic function of CXCL12 facilitates the mobilization and migration of osteoblast progenitor cells to tissue defects, thereby accelerating bone regeneration [13]. Besides its pro-regenerative effects, CXCL12 also contributes to the creation of a pro-inflammatory environment. Thus, the CXCL12/CXCR4 axis is associated with various infectious and inflammatory diseases, including osteoarthritis (OA) and rheumatoid arthritis (RA) [14–18].

OA is the most common joint disease and characterized by progressive cartilage degeneration, subchondral sclerosis, chronic synovial inflammation, and osteophyte formation [19]. At the molecular level, OA progression is orchestrated by a complex network of pathomechanisms and mediators, comprising reactive oxygen species, proteolytic matrix metalloproteinases (MMPs), cytokines, and chemokines. In case of the latter, CXCL12 has been described as a potential driver of OA. The chemokine is secreted by fibroblast-like synoviocytes (FLS) upon synovial inflammation and is highly associated with disease severity of OA and RA [17, 18]. Accordingly, synovial concentrations of CXCL12 were significantly elevated in OA patients (250 ± 26 ng/mL) and RA patients (750 ± 80 ng/mL) as compared to healthy individuals (70 ± 5 ng/mL) [20]. Besides the production by FLS, CXCL12 might also derive from the subchondral bone which undergoes aberrant remodeling during OA progression [21]. Both receptors, CXCR4 and ACKR3, are expressed on chondrocytes. However, most studies focus on the CXCL12/CXCR4 interaction. One possible explanation is that ACKR3 expression remains unaltered in OA [22], whereas CXCR4 has been reported to be upregulated in OA cartilage [15, 20]. Nevertheless, Li et al. recently postulated that the CXCL12/ACKR3 interaction might also be involved in the pathogenesis of OA [23].

In case of CXCR4, there is evidence that binding of CXCL12 promotes a hypertrophic phenotype in chondrocytes, characterized by enhanced catabolic enzyme production (e.g., matrix metalloproteinase 13 (MMP-13)) and collagen type X expression [24]. In line with this, inhibition of the CXCL12/CXCR4 signaling by the small molecule antagonist AMD3100 (Plerixafor, Mozobil) was reported to attenuate spontaneous OA development in Dunkin Hartley guinea pigs [15], as well as both, surgically-induced OA [25] and collagen-induced arthritis (CIA) in mice [26]. In contrast, current data suggest a protective role of CXCR4 in surgically-induced OA as demonstrated in a chondrocyte-specific, conditional knockout mouse model and after injection of CXCR4-overexpressing human cartilage-derived progenitor cells (hCPCs) in rabbits [27, 28]. Moreover, protective effects of CXCR4 inhibition conflict with the pro-regenerative features of the pathway. It should be further considered that the most commonly used inhibitor in these studies, AMD3100, acts as a weak allosteric agonist of ACKR3 [29].

To date, three CXCR4 antagonist have been approved for clinical applications [30–32]. However, these ligands are only approved for autologous stem cell transplantation in Non-Hodgkin’s lymphoma and the treatment of the WHIM syndrome, a rare congenital immunodeficiency disorder. One decade ago, another promising CXCR4 inhibitor was identified – the Endogenous Peptide Inhibitor of CXCR4, termed EPI-X4 [33]. EPI-X4 is a 16 amino acids long endogenous fragment of human serum albumin and specifically binds to CXCR4 thereby acting as an antagonist and inverse agonist [33]. Using rational drug design, EPI-X4 was optimized for its interaction with CXCR4 resulting on the derivative EPI-X4 JM#21 (ILRWSRKLPCVS). This optimized derivative demonstrated superior antagonistic activities compared to the wild type peptide and was successfully tested in different cancer models [34–36], as well mouse models of atopic dermatitis and eosinophilic asthma [37]. Notably, in both inflammatory mouse models AMD3100 was only poorly active. Thus, EPI-X4 JM#21 could represent a promising therapeutic option for the treatment of CXCR4-associated chronic diseases.

To shed further light on the role of CXCL12-mediated effects after binding to CXCR4 and ACKR3 in chondrocyte behavior, we investigated the expression of the chemokine and its receptors under different conditions, such as cartilage trauma, inflammation, and senescence. Further, we tested the therapeutic effects of the CXCR4 inhibitors EPI-X4 and its derivative EPI-X4 JM#21 on viability, migration, proliferation, and differentiation of human chondrocytes.

## Methods

### Peptide synthesis

EPI-X4 and its optimized derivative EPI-X4 JM#21 were synthesized as described previously [37]. Briefly, the peptides were synthesized via standard Fmoc solid-phase peptide synthesis using a Liberty Blue microwave synthesizer (CEM Corporation, Matthews, NC, USA) and then purified using reversed-phase high-performance liquid chromatography (Waters, Milford, MA, USA), employing an acetonitrile/water gradient under acidic conditions on a Phenomenex C18 Luna column (5 µm particle size, 100 Å pore size). Purified peptides were lyophilized on a freeze-dryer (Labconco, Kansas City, MI, USA), and the molecular mass was verified by liquid chromatography–mass spectrometry (LC–MS; Waters, Milford, MA, USA). The peptides were dissolved in dimethyl sulfoxide (DMSO, Sigma-Aldrich, Hamburg, Germany) at a stock concentration of 3 mM and further diluted in phosphate-buffered saline (PBS). AMD3100 octahydrochloride hydrate (#A5602) was purchased from Sigma-Aldrich, Hamburg, Germany and dissolved in H_2_O to create a 10 mM stock. Human and mouse CXCL12 were purchased from Peprotech, Hamburg, Germany (#300-28A) and dissolved in H_2_O at a concentration of 100 μg/mL.

### Human cartilage tissue

Clinical samples were obtained from donors undergoing knee replacement surgery. All donors provided their written informed consent in accordance with the guidelines of the Ethics Committee of the University of Ulm following the instructions of the Declaration of Helsinki (ethical approval No. 353/18). In this study, we used cartilage from a total of 47 donors (23 male, 24 female) with a mean age of 69.0 years (SD: 10.25).

### Preparation, traumatization, and cultivation of cartilage explants

Full-thickness cartilage explants were isolated with a biopsy punch (∅ 6 mm) from macroscopically intact (Osteoarthritis Research Society International (OARSI) grade ≤ 1 [38]) and highly degenerated cartilage tissue (OARSI ≥ 3). Explants assigned for RNA isolation or histological analysis were either immediately snap-frozen in liquid nitrogen or 4% formalin.

Only cartilage explants extracted from macroscopically intact tissue were used for ex vivo experiments. Explants were cultured in serum-free medium [SFM: DMEM (Live Technologies, Paisley, UK), 1 g/L glucose, 1% pyruvate (Sigma-Aldrich, Darmstadt, Germany), 1% non-essential amino acids (Bio-Sell, Feucht, Germany), 0.5% L-glutamine (PAN Biotech, Aidenbach, Germany), 0.5% penicillin/streptomycin (PAN Biotech), 40 μM 2-phospho-L-ascorbic acid trisodium salt (Sigma-Aldrich), 0.1% Insulin-Transferrin-Selenium (Live Technologies)] at 37 °C, in 5% CO_2_, and 95% humidity. A single impact of 0.59 J was applied on cartilage explants using a drop tower as previously described [39, 40].

During running experiments, explants were stimulated for 7 days with 200 ng/mL CXCL12 and either 1 µM peptides (EPI-X4 or EPI-X4 JM#21), or 1 µM AMD3100.

### Isolation and cultivation of human articular chondrocytes (hAC)

Solely macroscopically intact cartilage tissue was used to isolate hAC. Tissue was at first digested with 0.2% pronase (Sigma-Aldrich) for 45 min, following by an overnight digestion with 0.025% collagenase (Sigma-Aldrich) at 37 °C. A 40 µm cell strainer was used to remove residual cartilage fragments and cells were cultured in basal medium [BM: 1:1 DMEM and Ham´s F12 (PAN Biotech), 10% fetal bovine serum (FBS; PAN Biotech), 1 g/L glucose, 0.5% L-glutamine, 0.5% penicillin/streptomycin, 40 μM 2-phospho-L-ascorbic acid trisodium salt]. hAC were split at a confluency of 80% and used in passage p1 – p3.

During experiments, hAC were cultured in medium with reduced FBS content (5%) and stimulated with 200 ng/mL CXCL12, 1 µM compounds and/or 10 ng/mL IL-1β. Stress-induced premature senescence (SIPS) was induced by Doxorubicin (Doxo) (Selleckchem) treatment. The senescent phenotype was confirmed as previously described [41–43]. In brief, hAC were exposed to 0.1 µM Doxo for 7 days. Doxo was refreshed three times per week.

### Gene expression analysis

To isolate total RNA from cryopreserved cartilage explants, a microdismembrate S (B. Braun Biotech, Melsungen, Germany) was applied to mince the tissue. Subsequently, RNA was extracted by means of the RNeasy Lipid Tissue Mini Kit (Qiagen, Hilden, USA). In an analogous manner, total RNA was extracted from cultured cells using the RNeasy Mini Kit (Qiagen). Next, reverse transcription was performed using the Omniscript RT Kit (Qiagen).

To quantify RNA expression of the individual genes, TaqMan Gene Expression Mastermix (Live Technologies) together with Assays (supplementary Table S1) was used for real-time polymerase chain reaction (StepOnePlus Real-Time PCR System, Live Technologies). mRNA expression levels were calculated with the ΔΔCt method relative to the reference samples (macroscopically intact cartilage tissue or untreated control) and *GAPDH* and *HPRT1* served as reference genes.

### Histological analysis

Immunohistochemistry (IHC) was performed with paraffin embedded samples which were cut in sections of 3.5 µm and dewaxed and rehydrated prior to the staining. In the case of CXCR4 and collagen type II, 1 mg/mL pepsin in 0.5 M acetic acid (30 min, 37 °C) was used for antigen retrieval. In case of ACKR3, sections were incubated in 10 mM citrate buffer overnight at 65 °C. Next, primary antibodies (collagen type II 1:100, AF5710, Acris, Herford, Germany; CXCR4 1:500, MAB172, R&D Systems, Minneapolis, USA; ACKR3 1:2000, PA3-069, Live Technologies) were applied overnight (4 °C) and the sections were subsequently incubated in 3% hydrogen peroxide (30 min). Afterwards, the LSAB2 System horseradish peroxidase kit (Dako, Hamburg, Germany) was applied and gill´s haematoxylin (Merck, Darmstadt, Germany) was used to visualize nuclei. At least 3 images (Axioskop 2 mot plus microscope (Carl Zeiss, Oberkochen, Germany)) of randomly selected sections were quantified by manual counting and the resulting percentage of positive cells was used for statistical analysis.

In the case of Safranin O staining, dewaxed and rehydrated sections were first stained with Weigert´s iron hematoxylin (Merck) to stain nuclei. Next, sections were stained with 0.03% Fast Green (Sigma-Aldrich,) followed by 0.1% Safranin O (Chroma, Köngen, Germany).

### Cytotoxicity/cell proliferation assay – alamarBlue assay

To quantitatively assess the cell proliferation and cytotoxicity of hAC, an alamarBlue (BioRad, Munich, Germany) assay was used. The assay is based on the conversion of non-fluorescent resazurin to fluorescent resorufin during cellular respiration. The fluorescence intensity of resorufin was measured at 550 nm excitation and 590 nm emission with a multimode microplate reader Infinite M200 Pro (Tecan, Crailsheim, Germany). Unstimulated cells served as baseline (100% cell viability).

### Live/dead viability/cytotoxicity assay

A Live/Dead Viability/Cytotoxicity Assay (Live Technologies) was applied to determine the number of living and dead cells in cartilage explants. A small tissue section of 0.5 mm thickness was extracted from an unfixed cartilage explant and incubated for 40 min in a staining solution (1 µM calcein and 2 µM ethidium homodimer-1). Microscopic analysis was carried out with a Axioskop 2 mot plus microscope and using a z-stack model (AxioVision software).

### Chondrogenic differentiation

Pellet cultures were generated containing 3.5 × 10^5^ hAC in passage 3 and were cultured in chondrogenic differentiation medium [SFM: DMEM, 4.5 g/L glucose, 1% L-glutamine, 1% penicillin/streptomycin, 200 μM 2-phospho-L-ascorbic acid trisodium salt, 0.1% Insulin-Transferrin-Selenium, 0.1 µM dexamethasone (Sigma-Aldrich), 40 µg/mL L-proline (Sigma-Aldrich), 1% pyruvate, 10 ng/mL rhTGF-β3 (PeproTech), 10 ng/mL rhBMP6 (PeproTech)]. The medium was changed twice per week and 200 ng/mL CXCL12 and 1 µM compounds was added. After 28 days, resulting cell pellets were embedded in paraffin and prepared for histology (Collagen type II-IHC and Safranin O staining). For evaluation, a previously established scoring system was used (supplementary Table S2) [44].

### Wound healing assay

At first, a 2 Well Culture-Insert (Ibidi, Gräfelfing, Germany) was attached on a well of a 24 well plate. In each well of the insert, 70 µL cell suspension containing 20,000 hAC (passage 1 or 2) was added and incubated overnight. Next, medium with reduced FBS content (5%) was added and the insert carefully removed. Cells were stimulated with 200 ng/mL CXCL12, 1 µM EPI-X4, 1 µM EPI-X4 JM#21 or 1 µM AMD3100. Images were captured (Canon EOS 700D camera (Canon, Krefeld, Germany)) after 0 h and 24 h. Cells migrated into the gap were manually counted as previously described [42, 45].

### Live cell imaging

To assess undirected migration of the cells, 30,000 hAC (passage 1 or 2) were seeded on a 24 well plate and stimulated the following day with 200 ng/mL CXCL12, 1 µM EPI-X4 JM#21 or 1 µM AMD3100. Subsequently, cell localization was monitored for 10 h with a Live Cell Imaging Microscope Leica DMI6000 I (Leica Microsystems, Wetzlar, Germany). Images were acquired every 30 min and at least 6 cells of each well were manually tracked with ImageJ 2.9.0. The average migration distance was calculated and values were normalized to the untreated control.

### Ki-67 immunocytochemistry (ICC)

hAC at passage 1 were seeded onto a CultureSlide (Corning, Glendale, USA) and stimulated the following day with 200 ng/mL CXCL12, 1 µM EPI-X4 JM#21 or 1 µM AMD3100. After 48 h, cells were fixated with 4% formalin, permeabilized with 0.1% TritonX100 (Merck), and a Ki-67 antibody (1:250, ab16667, Abcam, Cambridge, UK) was added overnight (4 °C). On the next day, an Alexa Fluor 488 labled anti-rabbit antibody (1:200, ab150077, Abcam) was added and nuclei were stained with DAPI (0.25 µg/mL). The fluorescence microscope Axioskop 2 mot plus was used for imaging and Ki-67 appeared in green and nuclei in blue.

### Osteogenic differentiation and evaluation

hAC were seeded in passage 2 at a density of 1 × 10^4^ cells/cm^2^ and osteogenic differentiation was induced on the next day by addition of osteogenic differentiation medium [ODM: DMEM, 10% FBS, 1 g/L glucose, 1% L-glutamine, 1% penicillin/streptomycin, 10 mM β-glycerophosphate disodium salt hydrate (Sigma-Aldrich), 0.1 µM dexamethasone, 200 µM 2-phospho-L-ascorbic acid trisodium salt]. During the cultivation period of 21 days, cells were stimulated with either 200 ng/mL CXCL12, 1 µM EPI-X4 JM#21 or 1 µM AMD3100. Medium was changed twice a week and additives were refreshed every time. Cells cultured in BM served as negative control.

Evaluation of osteogenic differentiation was carried out by means of an Alizarin Red S staining which indicates calcium deposition. At first, cells were incubated in 70% ethanol for 1 h and subsequently stained with a 40 mM Alizarin Red S solution. For quantification, the cells were washed carefully and bound Alizarin Red S was dissolved in 10% Cetylpyridinium (Sigma-Aldrich). With a multimode microplate reader Infinite M200 Pro (Tecan Austria GmbH, Crailsheim, Germany), absorbance at 652 nm was measured and the amount of Alizarin Red S was calculated based on a standard calibration curve.

### ß-arrestin recruitment

Fifteen thousand HEK293T cells were seeded in 92 µL complete DMEM medium (supplemented with 10% FCS, 1% L-Glutamine and 1% penicillin/streptomycin) and allowed to adhere overnight. On the next day cells were transfected with plasmids containing a β-arrestin-2 coupled to a SmBiT and a CXCR4 or ACKR3 construct coupled to the LgBiT of the NanoBiT protein–protein-interaction assay system. The SmBiT was coupled to the intracellular C-term of the receptor, while the LgBiT was coupled to the N-term of the ß-arrestin. For the transfection, LT1 transfection reagent was used according to manufacturer’s instructions. The next day, medium was changed to 100µL pre-warmed OptiMEM before luminescence measurements were initiated.

For antagonism measurements, cells were first supplied with compounds and 10 min later with 30 nM CXCL12. Subsequently, luminescence signal was measured over 1 h. Baseline luminescence of each well in the first 10 min was averaged and used to calculate the signal-to-noise ratio for all subsequent time points of the same well. The signal-to-noise ratios were further recalculated as a ratio to the average of buffer-only treated wells. These values were plotted over time and areas under curve (AUCs) were determined for each well. Data were normalized to buffer-only wells as 0% and CXCL12-only treated wells as 100%.

For agonism measurements, single compounds were added to the wells after 10 min of background measurement. Baseline correction was performed in the same manner and resulting AUCs were compared to the buffer-only control wells to calculate n-fold ß-arrestin induction.

### Statistical analysis

Experiments were performed at least in triplicates with cartilage samples or cells derived from different donors (biological replicates) and Graph Pad Prism, version 10.4.1 was used for statistical analysis (significance level p < 0.05). Results are presented as box-and-whiskers plots including all data points and the Shapiro–Wilk test was applied to test for normal distribution. Normally distributed data was analyzed with an unpaired or paired t test and one-way ANOVA and for not normally distributed data, Friedman test and Wilcoxon signed-rank test was used.

## Results

### CXCR4 expression in chondrocytes is highly upregulated during cartilage degeneration, inflammation, and senescence

**Fig. 1:**
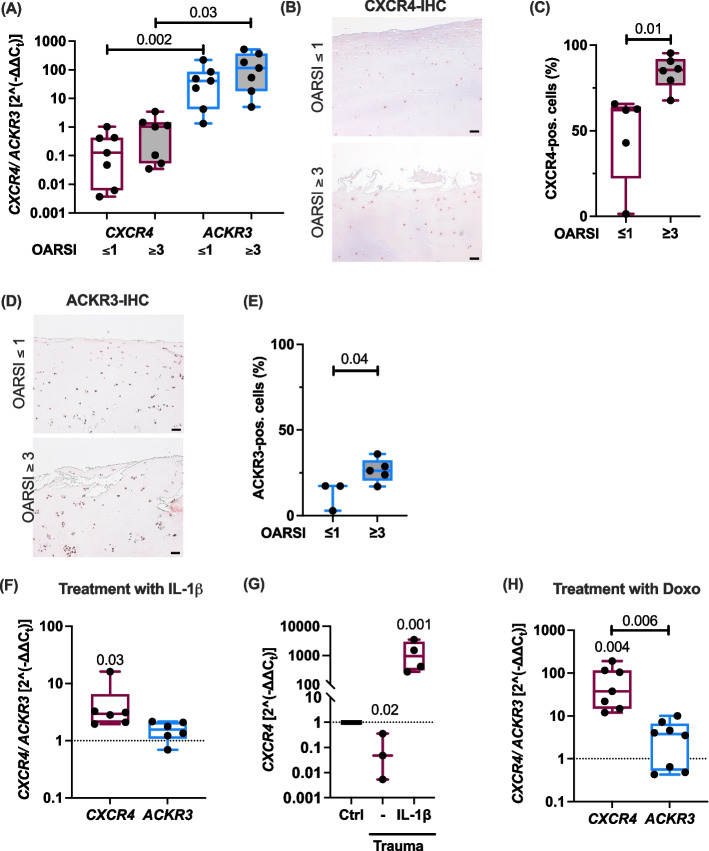
Expression of CXCR4 and ACKR3 by hAC and in cartilage under pathophysiologic conditions. **A** Gene expression of *CXCR4* and *ACKR3* in macroscopically intact (OARSI ≤ 1) and highly degenerated (OARSI ≥ 3) human cartilage. Friedman test; *n =* 7. IHC staining of (**B**) CXCR4 and (**D**) ACKR3 in cartilage of different OARSI grades (Scale bars equal 50 µm) and (**C**, **E**) the corresponding quantification. Unpaired t test; n ≥ 3. Gene expression of *CXCR4* and *ACKR3* in (**F**) hAC exposed to 10 ng/mL IL-1β for 24 h (Wilcoxon test; *n =* 6), (**G**) after cartilage trauma with or w/o addition of IL-1β (paired t test; n ≥ 3), and (H) in Doxo-treated chondrocytes (one-way ANOVA; n ≥ 7). Untreated cells or cartilage (in G) served as controls

First, we investigated the expression of CXCR4 and ACKR3 in human cartilage. Although, the gene expression of *CXCR4* was only increased by trend in cartilage of different OA grades, we confirmed an enhanced expression of CXCR4 in highly degenerated OA cartilage (OARSI ≥ 3) as compared to macroscopically intact tissue (OARSI ≤ 1) by means of IHC (Fig. 1A-C). In contrast, the expression levels of ACKR3 were only slightly elevated (Fig. 1D,E). Moreover, we identified the pro-inflammatory cytokine IL-1β as strong inducer of *CXCR4* in isolated chondrocytes (Fig. 1F) and after ex vivo cartilage trauma (Fig. 1G). However, ex vivo trauma alone even decreased the gene expression of *CXCR4* (Fig. 1G). We previously reported that cartilage trauma leads to the release of damage-associated molecular patterns, causing a pro-inflammatory response of FLS [46]. In accordance with this, we found a significant increase in *CXCL12* gene expression at 4 days after stimulation of FLS with trauma-conditioned medium (supplementary Fig. S3A). Furthermore, senescent chondrocytes exhibited significantly higher *CXCR4* mRNA levels as demonstrated in a Doxo-based in vitro SIPS model (Fig. 1H). Accordingly, senolytic therapy using Dasatinib and Quercetin, as previously described [47], reduced CXCR4 gene expression (supplementary Fig. S3B). Overall, *CXCR4* expression in chondrocytes was upregulated under different pathophysiologic conditions, while *ACKR3* remained largely unaffected.

### Activation or inhibition of the CXCL12/CXCR4 axis has no influence on cell viability or hypertrophy in chondrocytes

**Fig. 2:**
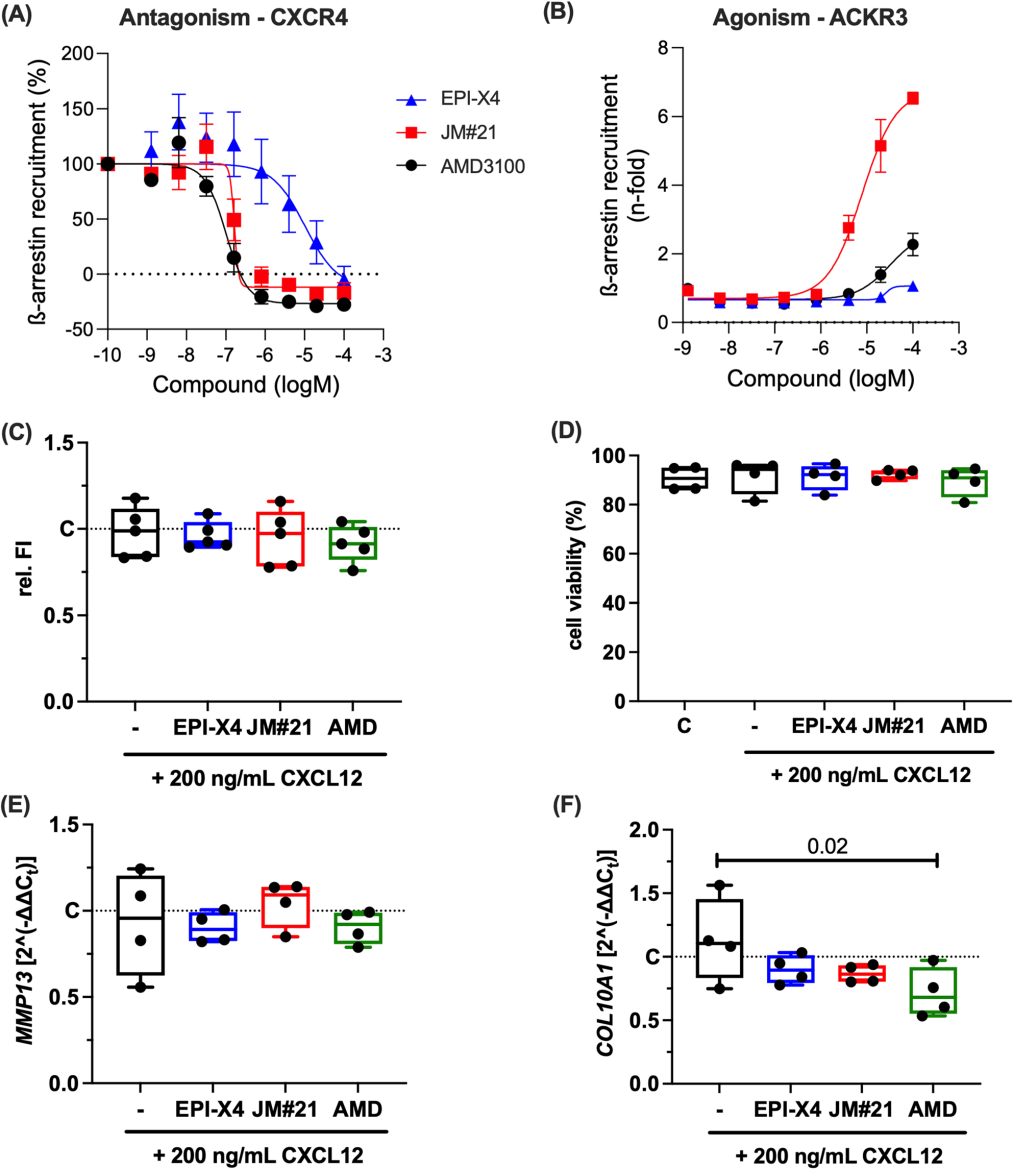
Effect of CXCR4 activation and inhibition on cell viability and expression of hypertrophy markers. **A** Inhibition of CXCL12-induced CXCR4 β-arrestin-2 recruitment. HEK293T cells were transfected with a plasmid containing β-arrestin-2 and a CXCR4 construct coupled to the two parts of the NanoBiT protein–protein interaction assay system. Cells were stimulated with 30 nM CXCL12 in the presence of compounds, and luminescence signals were recorded over time. Shown are the areas under the curves normalized to the buffer control. **B** ACKR3 β-arrestin recruitment. Transfected HEK293T cells were treated with the compounds and signals determined as described above. **C** Relative fluorescence intensity (FI) of the alamarBlue assay at 24 h after stimulation of isolated hAC with CXCL12 with or without addition of the CXCR4 inhibitors [1 µM]; *n =* 5. **D** Quantification of the live/dead assay of cartilage at day 7 after stimulation with CXCL12 with or without addition of the CXCR4 inhibitors [1 µM]; *n =* 5. Gene expression analysis of (**E**) *MMP13* and (**F**) *COL10A1* (one-way ANOVA) in isolated hAC at 48 h after stimulation with CXCL12 with or without addition of the CXCR4 inhibitors [1 µM]; *n =* 4

Here, we compared the effects of the small molecule CXCR4 antagonist AMD3100 with two CXCR4-targeting peptides, EPI-X4 and its derivative EPI-X4 JM#21. All compounds inhibited CXCL12-induced recruitment of β-arrestin-2 at CXCR4, with IC_50_ values of 101.6 nM (AMD3100), 10.17 nM (EPI-X4), and 163.6 nM (EPI-X4 JM#21), as expected [48] (Fig. 2A). In addition, we confirmed the weak agonistic activity of AMD3100 to recruit β-arrestin-2 after ACKR3 interaction (Fig. 2B) [48]. The wild type peptide EPI-X4 did not activate ACKR3, even at concentrations up to 10 µM (Fig. 2B). In contrast, the optimized derivative EPI-X4 JM#21 led to β-arrestin-2 recruitment at ACKR3 with an EC_50_ value of 7.8 µM (Fig. 2B), and is thus an antagonist for CXCR4 and an agonist for ACKR3.

**Fig. 3:**
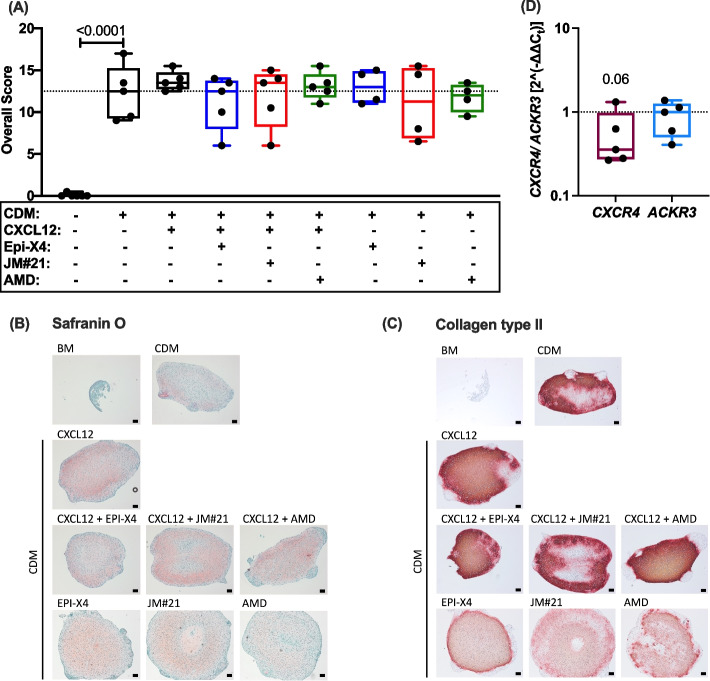
Influence of the CXCL12/CXCR4 pathway on chondrogenic re-differentiation of hAC. **A** Scoring of the chondrogenic re-differentiation of isolated hAC in presence and absence of CXCL12 and different CXCR4 inhibitors [1 µM] after 28 d. One-way ANOVA; n ≥ 4. Representative staining of (**B**) glycosaminoglycans with Safranin O and (**C**) collagen type II with IHC. All images were taken from the same donor, indicated in orange in (**A**). Scale bars equal 100 µm. **D** Gene expression of *CXCR4* and *ACKR3* in hAC after chondrogenic re-differentiation at day 28. Unpaired t test; *n =* 5. BM = basal medium; CDM = chondrogenic differentiation medium

It was previously observed that CXCL12/CXCR4 interaction induces necrosis-dependent death in chondrocytes [49]. In our experiments, we did not observe any cytotoxic effects in isolated chondrocytes (Fig. 2C) or native cartilage tissue (Fig. 3D) exposed to 200 ng/mL CXCL12, which is equivalent to the synovial concentrations reported in OA patients [20]. Moreover, we could not observe any cytotoxic effects of the CXCR4 inhibitors – neither in combination with CXCL12, nor alone (Fig. 2C,D, supplementary Fig. S4). Although CXCL12 is thought to induce a hypertrophic phenotype in chondrocytes, addition of CXCL12 did not significantly induce the gene expression of *MMP13* and *COL10A1* in isolated chondrocytes (Fig. 2E,F).

### The CXCL12/CXCR4 axis does not play a decisive role in in vitro re-differentiation of chondrocytes

Isolated hAC are known to progressively lose their chondrogenic phenotype during in vitro conditions within three to four passages. The “dedifferentiated” hAC can be re-differentiated into mature chondrocytes in a 3D pellet culture model and stimulation with CDM for four weeks [43]. To investigate the potential influence of CXCL12 and the CXCL12/CXCR4 pathway on the re-differentiation process, we added the chemokine and the respective inhibitors during the in vitro chondrogenesis. Histologic assessment of the pellet culture did not reveal any significant effect of CXCL12 or the CXCR4 inhibitors on neocartilage formation after four weeks (Fig. 3A-C). However, the expression of CXCR4 was reduced by trend upon in vitro chondrogenesis, while that of ACKR3 remained stable (Fig. 3D, supplementary Fig. S5). This finding complements the observation of enhanced CXCR4 levels in highly degenerated cartilage and under pathophysiologic conditions as described above.

### CXCR4 inhibition reduces CXCL12-mediated chondrocyte migration but has no effect on proliferation

**Fig. 4:**
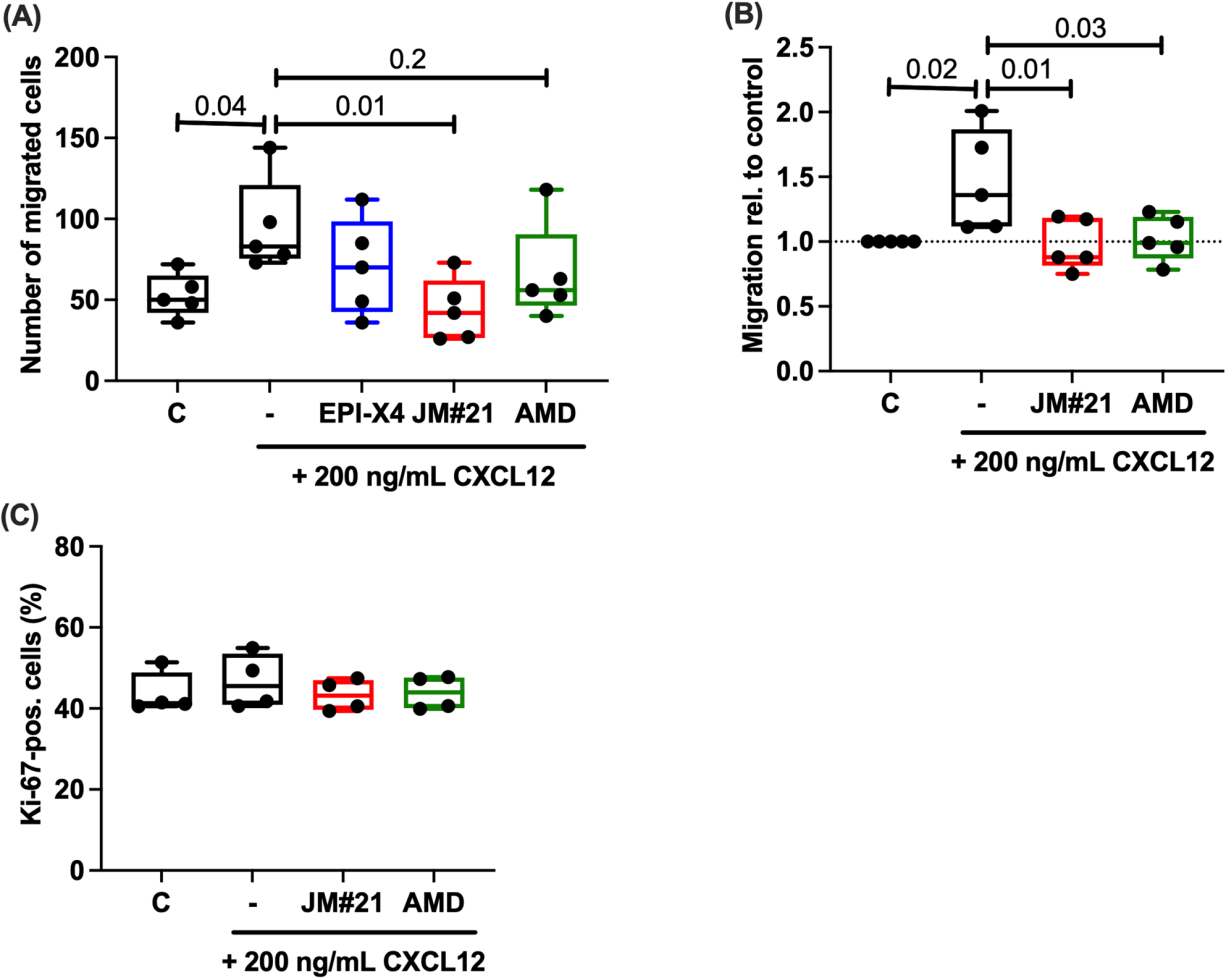
Influence of CXCL12 on hAC migration and proliferation. **A** Quantification of hAC in a wound healing assay in the presence and absence of CXCL12 and different CXCR4 inhibitors (1 µM) after 24 h. One-way ANOVA; n ≥ 5. **B** Migration distance of hAC in presence and absence of CXCL12 and different CXCR4 inhibitors [1 µM] after 48 h, determined by live-cell imaging. One-way ANOVA; *n =* 5. **C** Quantification of Ki-67-positive cells after 48 h of stimulation with CXCL12 and different CXCR4 inhibitors [1 µM]; *n =* 4

Stimulation with CXCL12 significantly increased the number of migrated hAC as demonstrated in a wound healing assay (Fig. 4A). Addition of CXCR4 inhibitors largely reversed the enhanced migration with EPI-X4 JM#21 exhibiting the strongest effects (Fig. 4A). Accordingly, the migration distance of isolated hAC was increased in the presence of CXCL12 and reversed in addition of the CXCR4 inhibitors as determined by live cell tracking (Fig. 4B). Owing to the high inter-donor variability in the migratory response to CXCL12, relative values are presented, while absolute values are provided in supplementary Fig. S6. Proliferation was not altered by CXCL12 stimulation or simultaneous addition of EPI-X4 JM#21 or AMD3100 (Fig. 4C,D).

### The CXCL12/CXCR4 axis does not play a decisive role in osteogenic differentiation of chondrocytes, despite enhanced levels of CXCR4

**Fig. 5:**
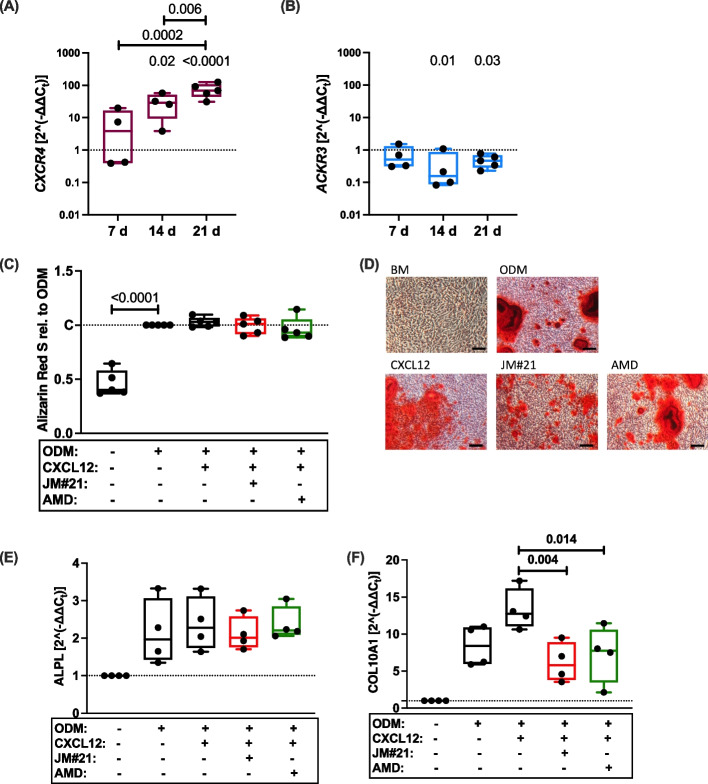
Influence of the CXCL12/CXCR4 axis on matrix calcification during hAC osteogenic differentiation. **A** Gene expression of (A) *CXCR4* and (**B**) *ACKR3* during osteogenic differentiation of hAC after 7 d, 14 d, and 21 d. One-way ANOVA; n ≥ 4. **C** Relative concentration of Alizarin Red S after osteogenic differentiation of hAC in the presence or absence of 200 ng/mL CXCL12 and different CXCR4 inhibitors (1 µM) after 21 d. One-way ANOVA; *n =* 5. **D** Representative images of the Alizarin Red S staining after osteogenic differentiation of hAC in presence and absence of 200 ng/mL CXCL12 and different CXCR4 inhibitors (1 µM) after 21 d. Scale bars equal 100 µm. Gene expression analysis of (**E**) ALPL and (**F**) COL10A1 in hAC after 14 d of osteogenic differentiation in hAC in the presence or absence of 200 ng/mL CXCL12 and different CXCR4 inhibitors (1 µM); *n =* 4. BM = basal medium; ODM = osteogenic differentiation medium

As CXCR4 and CXCL12 have been linked to chondrocyte hypertrophy, fracture healing, and osteogenesis [24, 50, 51], we investigated their role during in vitro osteogenesis of chondrocytes. In fact, we observed a time-dependent increase in *CXCR4* expression at 7, 14, and 21 days of in vitro osteogenic differentiation (Fig. 5A). In contrast, the gene expression of *ACKR3* was suppressed during osteogenic differentiation of hAC (Fig. 5B). However, neither the addition of CXCL12, nor inhibition of CXCR4 by EPI-X4 JM#21 or AMD3100 had any significant effect on matrix calcification (Fig. 5C,D). While gene expression analysis of ALPL (Fig. 5E), RUNX2, SP7, and IBSP (supplementary Fig. S7) after 14 d of osteogenic differentiation revealed no clear impairment of the ossification process in the presence of CXCR4 inhibitors, expression of the hypertrophic marker COL10A1 was significantly reduced in hACs treated with EPI-X4 JM#21 or AMD3100 (Fig. 5F).

## Discussion

Despite scientific evidence that the CXCL12/CXCR4 axis is involved in chondrocyte hypertrophy and cartilage homeostasis, its potential role in the pathogenesis of OA remains controversially discussed. In the present study, we elucidated the expression of CXCR4 in hAC and confirmed that the receptor was highly expressed under pathophysiologic conditions and processes (e.g., cartilage degeneration progression, IL-1β exposure, and chondrosenescence), while in vitro chondrogenesis reduced its expression. In contrast, ACKR3, was found comparably higher expressed in hAC and was only regulated during in vitro osteogenesis, where it was reduced by trend.

The increased expression of the CXCR4 on hAC under pathophysiologic conditions implies a higher susceptibility of the hAC towards CXCL12. Ex vivo cartilage trauma did not induce CXCR4 expression, despite promoting other OA-related processes such as cartilage degeneration, chondrocyte senescence, and regulated cell death, as previously described [40, 42, 52]. DAMP release following mechanical impact induced the expression of CXCL12 and other cytokines, including IL-1β, in FLS [42, 46], suggesting posttraumatic activation of the CXCL12/CXCR4 pathway in a paracrine manner. As the expression of ACKR3 remains unaffected, while the production of CXCL12 increases during OA progression, the chemokine might preferably bind to CXCR4. Although we were unable to confirm CXCL12-driven hypertrophy in vitro, the enhanced CXCL12/CXCR4 interaction may contribute to aberrant chondrocyte behavior during OA progression in vivo.

To our knowledge, this is the first report of enhanced CXCR4 expression in senescent hAC. Moreover, we observed that the gene expression of CXCL12 was significantly reduced in senescent cells of various tissues, including cartilage (hAC), synovial membrane, and bone (human primary osteoblasts) (data not shown). This finding was unexpected because the senescent cells typically express high levels of cytokines (e.g., IL-6 and GDF-15) and chemokines (e.g., IL-8/CXCL8 and Gro-a/CXCL1) [41, 42, 47]. This finding could explain the impaired regenerative potential of bone during aging due to the accumulation of senescent cells and warrants further investigation.

Although CXCR4 was strongly induced during osteogenic differentiation in hAC, we could not find any influence on Ca^2+^ deposition or gene expression of osteogenic markers by fueling (addition of CXCL12) or inhibiting (addition of EPI-X4 JM#21 or AMD3100) the CXCL12/CXCR4 pathway. However, during osteogenic induction, the hypertrophy marker COL10A1 tended to increase in the presence of CXCL12 and significantly reduced upon addition of the CXCR4 inhibitors EPI-X4 JM#21 and AMD3100. Moreover, CXCL12 increased the migratory activity in hAC in a CXCR4-dependent manner, but the involvement of the CXCL12/CXCR4 interaction in chondrocyte proliferation, or death was not confirmed and the pro-hypertrophic effect not striking in this in vitro study. Although the observed reduction in COL10A1 expression did not affect osteogenic differentiation in vitro, it might be more pronounced in vivo. In vivo, the CXCL12/CXCR4 axis plays a crucial role in chondrocyte hypertrophy and thus endochondral ossification. Accordingly, both CXCR4 and its ligand CXCL12 are highly expressed in hypertrophic chondrocytes in the growth plate [24] and were described as essential during bone formation and fracture healing [29, 53, 54]. In line with that, we observed a strong induction of CXCR4 expression during in vitro osteogenic differentiation of hAC. As the inhibition of CXCR4 did not affect mineralization during osteogenic differentiation and in consideration of the current literature, we assume that the pro-hypertrophic or pro-osteogenic effect strongly depends on the context. In vivo, for example, recruitment of progenitor cells and neovascularization are essential processes in bone formation, which are less pivotal during in vitro osteogenic differentiation. Both stem/progenitor cell migration and vascularization are demonstrably regulated by CXCL12 [50, 55]. Further, it should also be considered that only very high concentrations of AMD3100 (400 µM) impaired the gene expression of osteogenic markers during in vitro osteogenesis of MSCs to a limited extend [51]. However, matrix calcification was not affected in this study and the dosage does not appear physiologically relevant, regarding the clinical peak plasma concentrations of ~ 1 μM [51, 56].

Although our data imply that the addition of CXCL12 or the inhibition of CXCL12/CXCR4 signaling modulated the migratory activity of hACs, the pathway had no effect on chondrogenic differentiation or cartilage integrity ex vivo—neither beneficial nor detrimental. Nevertheless, we still assume that the CXCL12/CXCR4 axis plays a decisive role in OA progression. It should also be considered that the pathophysiological mechanisms are not mediated by chondrocytes alone. Qin et al. described that specific inhibition of the CXCL12/CXCR4 axis in the subchondral bone, via an osmotic pump, attenuates cartilage degeneration in a surgically-induced OA mouse model [57]. They demonstrated that increased CXCL12 levels in the subchondral bone primarily promoted bone deterioration due to aberrant MSCs recruitment and excessive osteoclast-driven bone resorption [57, 58]. As a consequence of the subchondral bone degeneration, osteoblast-derived CXCL12 is released to the cartilage and subsequent CXCL12/CXCR4 interaction on chondrocytes results in cartilage degradation. Therefore, inhibition of CXCL12/CXCR4 interaction in subchondral bone with AMD3100 reduced the severity of surgically-induced OA by stabilization of the subchondral bone microarchitecture [57]. Furthermore, CXCL12 not only induced abnormal osteoid islet formation in subchondral bone during OA development, but also promoted angiogenesis and subsequent subchondral bone innervation, which contributes to pain in progressed OA [59]. In line with the in vivo studies on knee OA, osteoblast-derived CXCL12 was found to enhance aberrant subchondral bone formation and exacerbate cartilage degeneration in a paracrine manner as observed in a rat overload-induced temporomandibular joint OA model. And again, subchondral bone deterioration occurred first, followed by cartilage damage [60]. Overall, these findings emphasize the substantial contribution of the subchondral bone and its crosstalk with articular cartilage during the pathogenesis of OA and indicate that the CXCL12/CXCR4 axis represents a central communication path between the tissues [57]. It is very likely that the therapeutic effects of AMD3100 observed in Dunkin Heartly guinea pigs, which spontaneously develop an early idiopathic OA, likewise result from the interference of the crosstalk between subchondral bone and cartilage [15].

The comparison between the novel CXCR4-targeted peptides EPI-X4 and EPI-X4 JM#21 and the FDA-approved CXCR4 inhibitor AMD3100 revealed that none of the candidates exhibited adverse effects on chondrocyte viability and differentiation into the chondrogenic or osteogenic lineage. Moreover, we observed that EPI-X4 JM#21 was more effective at impeding CXCL12-induced cell migration as compared to EPI-X4 and AMD3100, despite strong induction of the ACKR3 signaling. ACKR3 has been described as essential cofactor in CXCL12/CXCR4-mediated cell migration [61], thus, we assume that even excessive activation of ACKR3 and subsequent ß-arrestin recruitment, as demonstrated by EPI-X4 JM#21, does not inevitably result in migration. Similar inhibition of CXCL12-induced cell migration by the EPI-X4 derivative was recently reported in migrating cancer cells [35]. Although our findings indicate that the tested inhibitors have no adverse effect on articular chondrocytes, CXCL12/CXCR4-mediated recruitment of stem/progenitor cells is considered crucial in terms of joint and bone regeneration [1–3, 13, 24, 50, 54]. An impaired activation of migratory hCPCs, which are thought to possess pro-regenerative potential after cartilage injury, might have detrimental consequences [45]. Accordingly, CXCR4-overexpressing hCPCs exhibited an enhanced migratory activity towards CXCL12, secreted by injured menisci. Intra-articular injection of the CXCR4-overexpressing hCPCs significantly reduced cartilage erosion and increased meniscus healing in a lapine surgically-induced OA model [28]. Therefore, additional in vivo studies are required to clarify the influence of the inhibitors on cartilage repair.

## Conclusions

Overall, we conclude that the CXCL12/CXCR4 axis represents a potential target during OA progression. Inhibition of this pathway may attenuate subchondral bone deterioration and subsequent cartilage degeneration. However, as the CXCL12/CXCR4 pathway seems essential for tissue repair after injury, its inhibition during the acute phase following joint injury could suppress pro-regenerative processes. Thus, a delayed application of CXCR4 inhibitors might prevent OA progression, which seems to be promoted by the chronic activation of the pathway. The optimal timing for the treatment remains to be determined. With regard to the high side effects of AMD3100, the EPI-X4 derivative EPI-X4 JM#21 might represent a promising candidate for future testing in an in vivo OA model.

## Supplementary Information

Supplementary Material 1

## Data Availability

All data generated or analysed during this study are included in this published article and its supplementary information files.

## Abbreviations

ACKR3: Atypical chemokine receptor 3
BMP-2: Bone morphogenetic factor 2
CXCL12: C-X-C motif chemokine 12
CXCR4: CXC chemokine receptor type 4
Doxo: Doxorubicin
EPI-X: Endogenous Peptide Inhibitor of CXCR4
FCS: Fetal calve serum
H_2_O: Water
hAC: Human articular chondrocytes
hCPCs: Human cartilage-derived progenitor cells
IHC: Immunohistochemistry
IL: Interleukin
MSCs: Mesenchymal stem cells
OA: Osteoarthritis
OARSI: Osteoarthritis Research Society International
ODM: Osteogenic differentiation medium
RA: Rheumatoid arthritis
SDF-1: Stromal cell-derived factor 1
SFM: Serum-free medium
SIPS: Stress-induced premature senescence
TGF-ß: Transforming growth factor β
