# Supplementary material for: Targeting the CXCL12/CXCR4 pathway by an optimized derivative or EPI-X4 preserves chondrocyte function and offers a novel therapeutic approach in rheumatic diseases
Source: Arthritis Res Ther. 2025 Dec 2;27:222. doi: 10.1186/s13075-025-03691-9 (PMC12670779; doi:10.1186/s13075-025-03691-9)
Supplement: Supplementary file 1 — Supplementary Material 1 [file 13075_2025_3691_MOESM1_ESM.docx]

**Supplementary Table S1: Used TagMan Gene Expression Assays and primers.**

| **Gene** | **Assay ID / primer sequence** |
| --- | --- |
| *COL10A1* | Hs00166657_m1 |
| *CXCL12* | Hs00171022_m1 |
| *CXCR4* | Hs00237052_m1 |
| *ACKR3* | Hs00604567_m1 |
| *GAPDH* | Hs02758991_g1 |
| *HPRT1* | Hs02800695_m1 |
| *MMP13* | Hs00942584_m1 |

**Supplementary Table S2: Assessment of neo-cartilage formation.** Adapted from Riegger et al. 2018. The scoring system uses the pellet size (diameter), collagen type II, safranin O staining intensity, cell-cell distance (matrix production), and cell morphology. The categories were classified from 0 (= evidence for failed chondrogenic differentiation) to 3-5 (=evidence for successful chondrogenic differentiation). The overall score was calculated by adding up all individual scores of the categories.

| **Scoring categories** | **Score** |
| --- | --- |
| **1) Pellet size (diameter)** |  |
| < 900 µm | 0 |
| > 900 µm | 1 |
| > 1200 µm | 2 |
| > 1500 µm | 3 |
| > 1800 µm | 4 |
| > 2200 µm | 5 |
| **2) Collagen type II/ Safranin O staining intensity** |  |
| No staining | 0 |
| Weak staining (> 5 % positive) | 1 |
| Scattered moderate staining (> 25 % positive) | 2 |
| Even moderate stain (> 50 % positive) | 3 |
| Even intensive stain (> 85 % positive) | 4 |
| **3) Cell-Cell distance (matrix production)** |  |
| High cell density; no matrix between cells | 0 |
| High cell density; little matrix between cells | 1 |
| Moderate cell density; little matrix between cells | 2 |
| Low cell density; extensive matrix between cells | 3 |
| **4) Cell morphology** |  |
| Condensed/necrotic/pycnotic bodies | 0 |
| Spindel/fibrous | 1 |
| Mixed spindle/fibrous and rounded/chondrogenic | 2 |
| Majority rounded/chondrogenic | 3 |

**
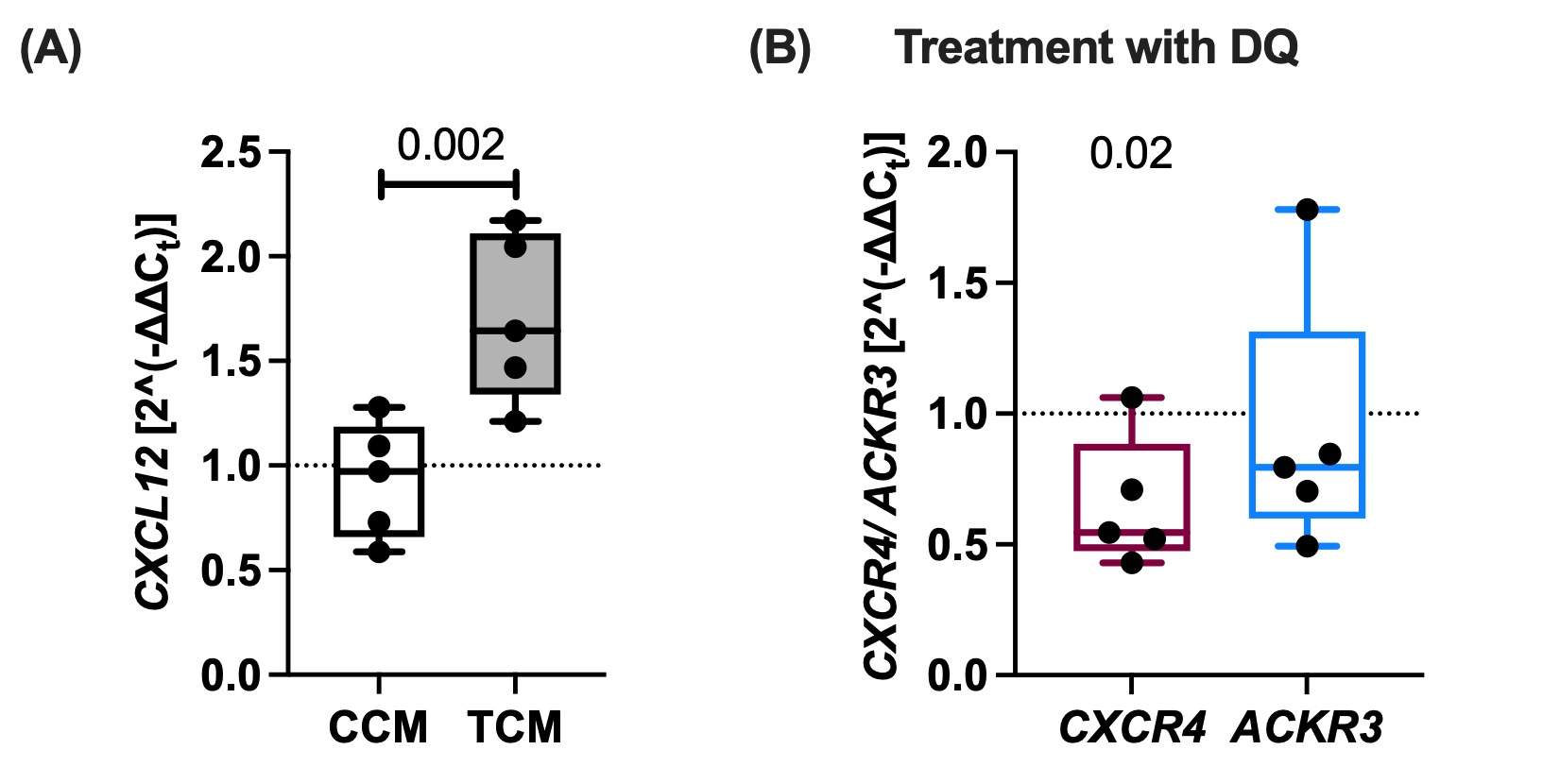
**

**Supplementary Figure S3:** (A) Gene expression of *CXCL12* in human fibroblast-like synoviocytes isolated from OA patients, treated with trauma-conditioned medium (TCM) for 4 days. TCM was collected 7 days after cartilage trauma. Cartilage conditioned medium (CCM) was collected from unimpacted cartilage explants and used as control. Paired t test; n = 5. (B) mRNA levels of *CXCR4* and *ACKR3* after senolysis. Human articular chondrocytes were treated with Dasatinib (250 mM) and Quercetin (5 µM) for 3 days, followed by a cultivation for 4 days without D+Q. Unpaired t test; n = 5.

**Supplementary Figure S4:** Relative fluorescence intensity (FI) of the alamarBlue assay at 24h after stimulation of isolated hAC with addition of the CXCR4 inhibitors [1 µM]; n = 5.


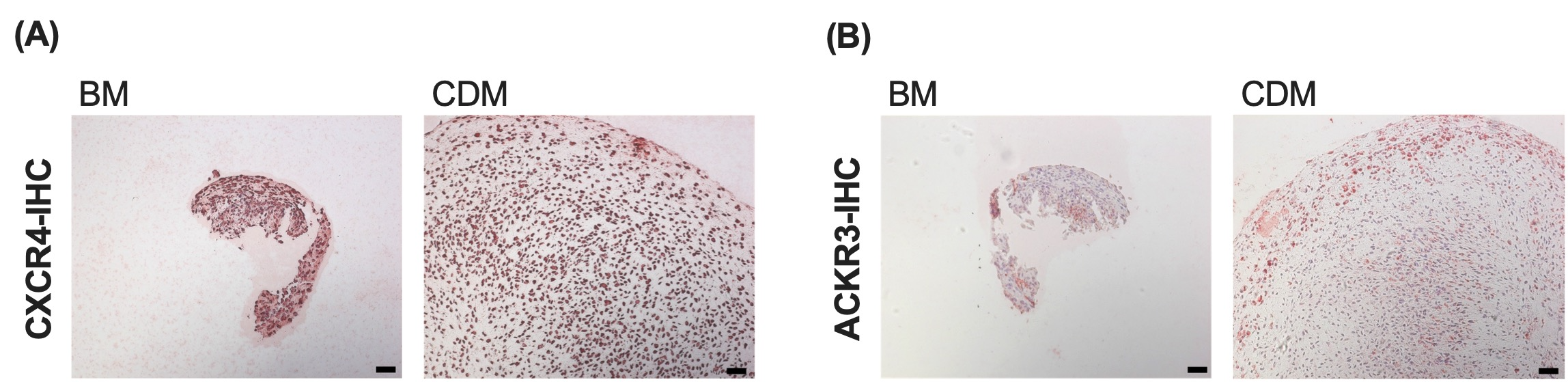


**Supplementary Figure S5:** Representative (A) CXCR4 and (B) ACKR3 staining of chondrogenic re-differentiation of isolated hAC by means of immunohistochemistry (IHC). Scale bars equal 50 µm. BM = basal medium; CDM = chondrogenic differentiation medium.

**Supplementary Figure S6:** Migratory activity of hAC stimulated with 200 ng/mL CXCL12 in the presence or absence of CXCR4 inhibitors [1 µM] assessed via live cell imaging given as absolute distance in µm.

**Supplementary Figure S7:** Gene expression analysis of (A) RUNX2, (B) SP7, and (C) IBSP in hAC stimulated with osteogenic differentiation medium (ODM) for 14d in presence or absence of 200 ng/mL CXCL12 and different CXCR4 inhibitors [1 µM]. hAC in basal medium served as undifferentiated control; n = 4.
